# Plastic antibodies tailored on quantum dots for an optical detection of myoglobin down to the femtomolar range

**DOI:** 10.1038/s41598-018-23271-z

**Published:** 2018-03-21

**Authors:** Ana Margarida Piloto, David S. M. Ribeiro, S. Sofia M. Rodrigues, Catarina Santos, João L. M. Santos, M. Goreti F. Sales

**Affiliations:** 1 BioMark/ISEP, School of Engineering of the Polytechnic Institute of Porto, Porto, Portugal; 2 LAQV/REQUIMTE, Faculty of Pharmacy of Porto University, Porto, Portugal; 3 EST Setúbal, CDP2T, Instituto Politécnico de Setúbal, Setúbal, Portugal; 4 CQE, Instituto Superior Técnico, Universidade de Lisboa, Lisboa, Portugal

## Abstract

A highly sensitive fluorescence detection probe was developed by tailoring plastic antibodies on the external surface of aqueous soluble quantum dots (QDs). The target was Myoglobin (Myo), a cardiac biomarker that quenched the intrinsic fluorescent emission of cadmium telluride (CdTe) QDs capped with mercaptopropionic acid (CdTe-MPA-QDs). The QDs were incubated with the target protein and further modified with a molecularly-imprinted polymer (MIP) produced by radical polymerization of acrylamide and bisacrylamide. The main physical features of the materials were assessed by electron microscopy, dynamic light scattering (DLS), UV/Vis spectrophotometry and spectrofluorimetry. The plastic antibodies enabled Myo rebinding into the QDs with subsequent fluorescence quenching. This QD-probe could detect Myo concentrations from 0.304 to 571 pg/ml (50.6 fM to 95 pM), with a limit of detection of 0.045 pg/ml (7.6 fM). The proposed method was applied to the determination of Myo concentrations in synthetic human serum. The results obtained demonstrated the ability of the modified-QDs to determine Myo below the cut-off values of myocardial infarction. Overall, the nanostructured MIP-QDs reported herein displayed quick responses, good stability and sensitivity, and high selectivity for Myo, offering the potential to be explored as new emerging sensors for protein detection in human samples.

## Introduction

Cardiovascular diseases are a global cause of deaths worldwide, accounting for nearly 17.3 million cases per year^1^. Monitoring cardiac protein biomarkers is vital to enable early detection of a myocardial infarction (MI) event. Myoglobin (Myo), creatine kinase (CK), CK isoenzyme MB (CK-MB) and cardiac troponins T and I (cTnT and cTnI), are examples of these biomarkers^2^. Although cTnT and cTnI are the gold standards for MI occurrence, Myo is the first biomarker released by the cell upon cardiac muscle injury^3^. Thus, simple and quick methods have to be addressed to act in the first stage of MI events.

In this regard, several methods have already been developed for Myo detection. These include the use of luminescence techniques that rely on immunoassays^4–6^, surface plasmon resonance (SPR)^7,8^, fluoroimmunoassay with fiber optics^9^, electrochemistry^10–16^ or immunoassays on magnetic-based platforms^17^. There are also several works reporting optical detection by using QDs as luminescent elements^18^ but mostly coupled to immune-based assays^19–25^. In general, immunoassays use natural antibodies, which are excellent probes in terms of selectivity and selectivity, leading these to current commercial applications as ELISA. However, these naturally based materials are expensive and require specific storage conditions.

An alternative to the above approaches replaces biological receptors by synthetic biomimetic materials, such as molecular imprinted polymers (MIPs), also known as plastic antibodies. MIPs are artificially designed 3D-materials, where the target biomolecule is molded by growing a polymeric structure around it. The 3D mold holds the capacity to rebind to the target molecule after its removal from the core polymeric structure^26^. These materials present several advantages over natural antibodies in immunoassays, such as improved chemical stability and sensitivity in aqueous buffers, expeditious synthesis, facile integration with transducers, and inexpensive costs of manufacture^27^.

Nevertheless, the preparation of plastic antibodies for proteins remains a challenge. A key element is the maintenance of the native conformation of the protein. Extreme pH and thermal conditions are commonly known to denature these biomolecules, resulting in the unfolding of their 3D structure with the subsequent loss of conformation^28^. A successful and simple approach in this context includes polymerization with acrylamide and bisacrylamide as monomers and cross-linker, making use of bulk or surface imprinting. These allow mild polymerization conditions and biocompatible environment to accommodate the protein upon rebinding. Several technical approaches for Myo imprinting reported include the use of Cryogels^29^, surface imprinting^30^, microcontact imprinting^31,32^, charged oriented imprinting^33^, electropolymerization^34^, grafting on silicon^35^ or graphite supports^36^ or using polymerizable liposomes^37^.

In turn, Quantum Dots (QDs) are nanostructured materials with large surface-to-volume ratio and size-correlated physical and chemical properties. The excellent properties frequently exhibited, such as high photoluminescence, chemical stability and good aqueous dispersibility, simplicity of preparation and tuning of optical properties, endow these new-generation luminescent materials with superior performances relative to conventionally used fluorescent organic dyes. QDs are used today in many fields^19,38–40^, including colorimetry^41^, photoelectrochemistry^42^ or electrochemistry^19,43^. To our knowledge there are few reports coupling QDs to MIPs^44^, with only three publications devoted to protein imprinting: haemoglobin^45^; bovine serum albumine^46^; and lysozyme, cytochrome C and methylated bovine serum albumin^47^. All these use a combination of QDs with silica material, either having the QDs as core or as core/shell, and none of these works target a Myo detection.

Thus, this works tries for the first time a combination of MIP and QDs for targeting Myo, combining the low cost and selective readings of MIPs and the sensitivity of QDs. Vinyl-based radical and non-covalent polymerization is employed herein instead of sol-gel chemistry. Vinyl-chemistry is inexpensive and simple, offering a wide range of monomer selection. Non-covalent imprinting is the most popular approach for protein imprinting among the last decades. In turn, sol-gel chemistry is harder to control and reproduce under the protein-restricted conditions of polymerization. Moreover, the vinyl polymer is tailored directly around the QD, yielding a very simple technological approach. Acrylamide-based monomers and cross-linkers are selected herein, because it yields an excellent matrix for protein recognition^33^, able to interact with the polar and hydrophilic groups of the protein. The type of polymerization, the strategies for protein removal, the time of incubation and the crosslinking density of the polymeric matrix are optimized. The optical readouts of MIP-QDs and its non-imprinted (NIP) counterparts, NIP-QDs, are monitored by fluorescence spectroscopy. Quantification of Myo was possible down to the femtomolar scale in synthetic human serum samples.

## Results and Discussion

### Physical and Chemical features of the raw QDs

#### Optical properties

**Figure 1:**
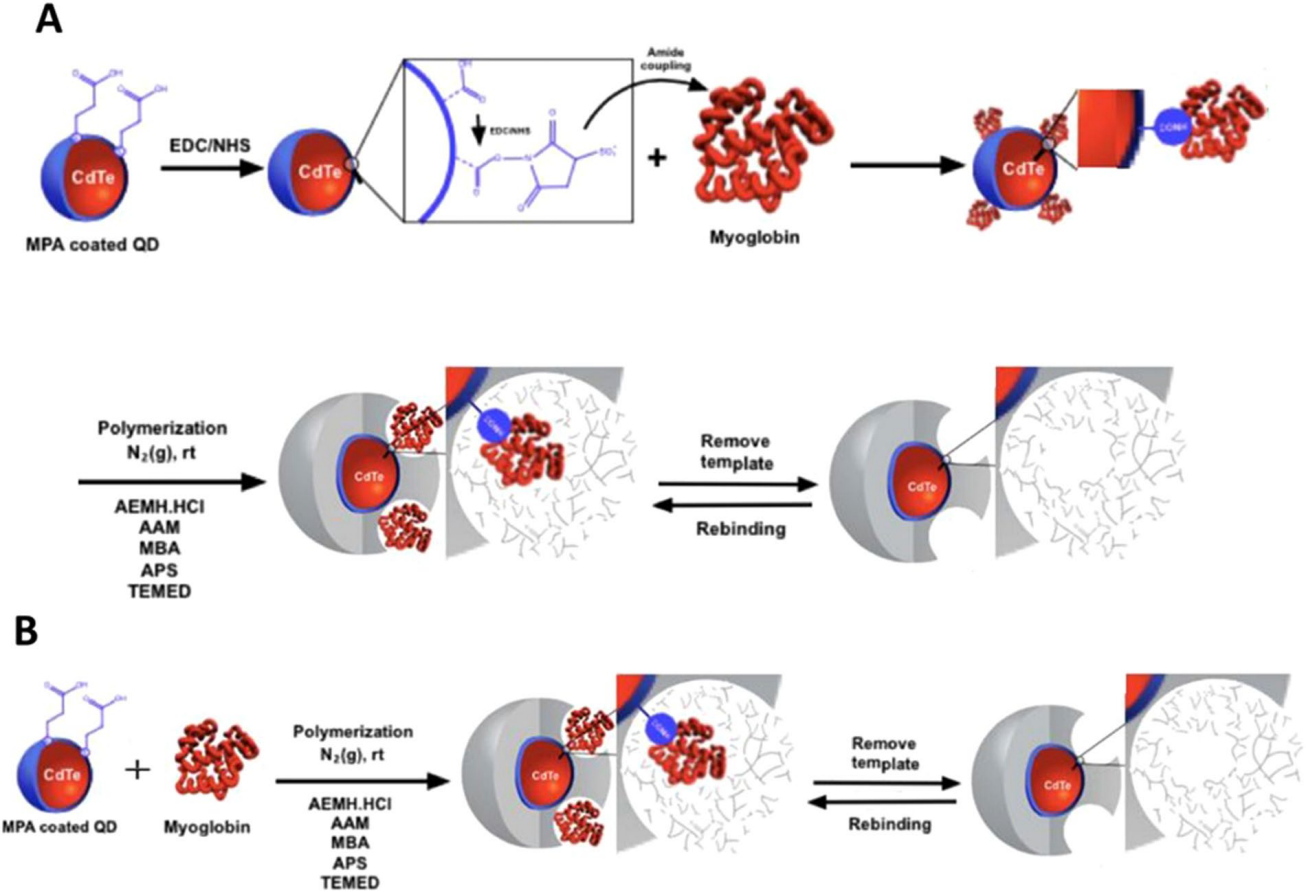
Schematic representation of (**A**) surface imprinting and (**B**) bulk imprinting strategies for the preparation of MIP-QDs.

**Figure 2:**
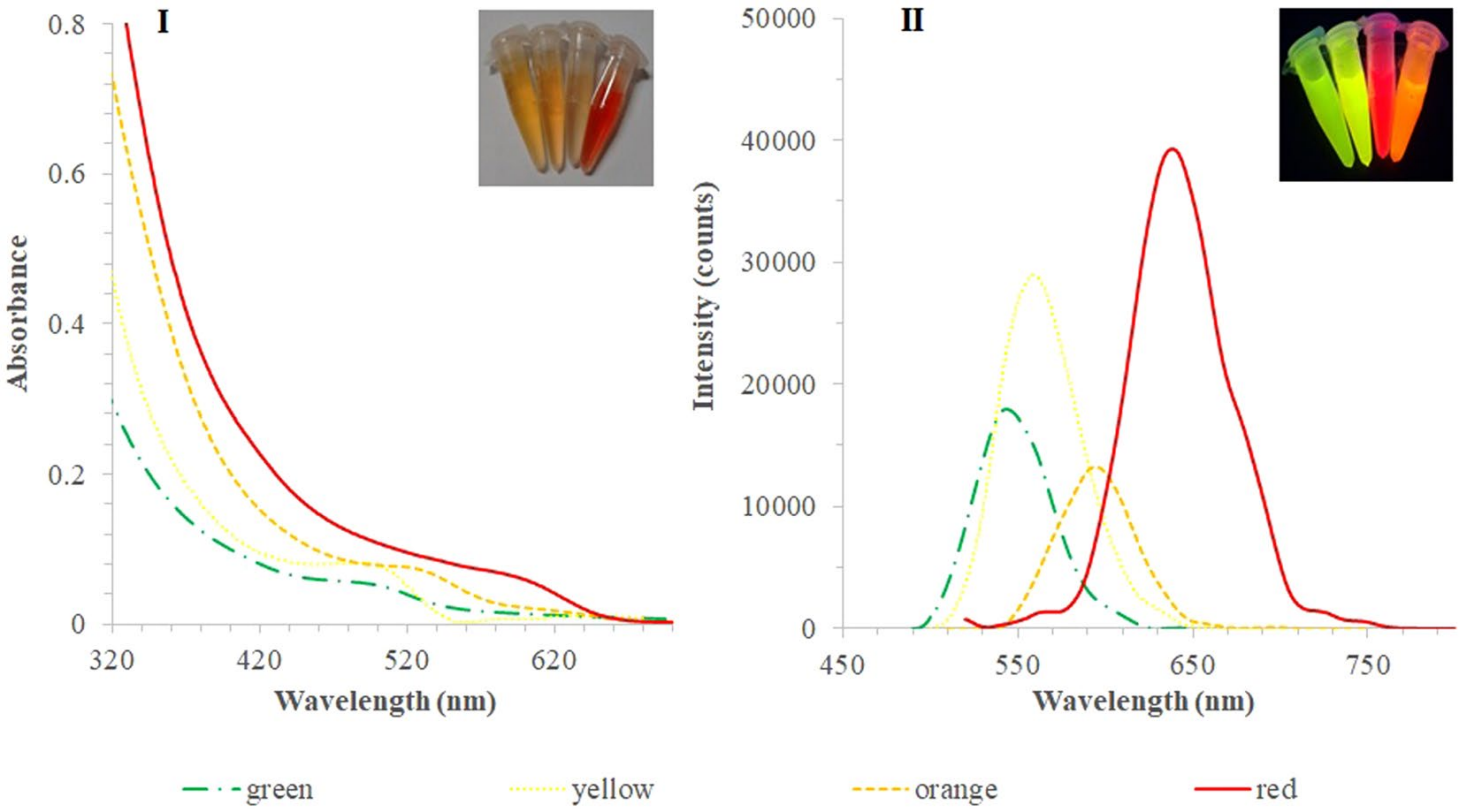
(**I**) UV-Vis absorption and (**II**) emission fluorescence spectra of raw QDs in PBS 10 mM. The average size of the QDs are (‒․-) 2.2 nm, (………․) 2.5 nm, (-----) 2.9 nm and (—) 3.7 nm (as estimated by equation 1).

The overall modification of the QDs depicted in Fig. 1 was established for different times, yielding different optical properties and size. The absorption and emission spectra of raw QDs dispersed in PBS 10 mM are shown in Fig. 2. As expected, a bathochromic shift was observed in the emission spectra with the increase of the reaction time of the QDs, which in turn was related to QDs of higher diameter. The red QDs recorded the highest fluorescence emission, in agreement with the absorption coefficient (Table S2).

#### Size and charge

**Figure 3:**
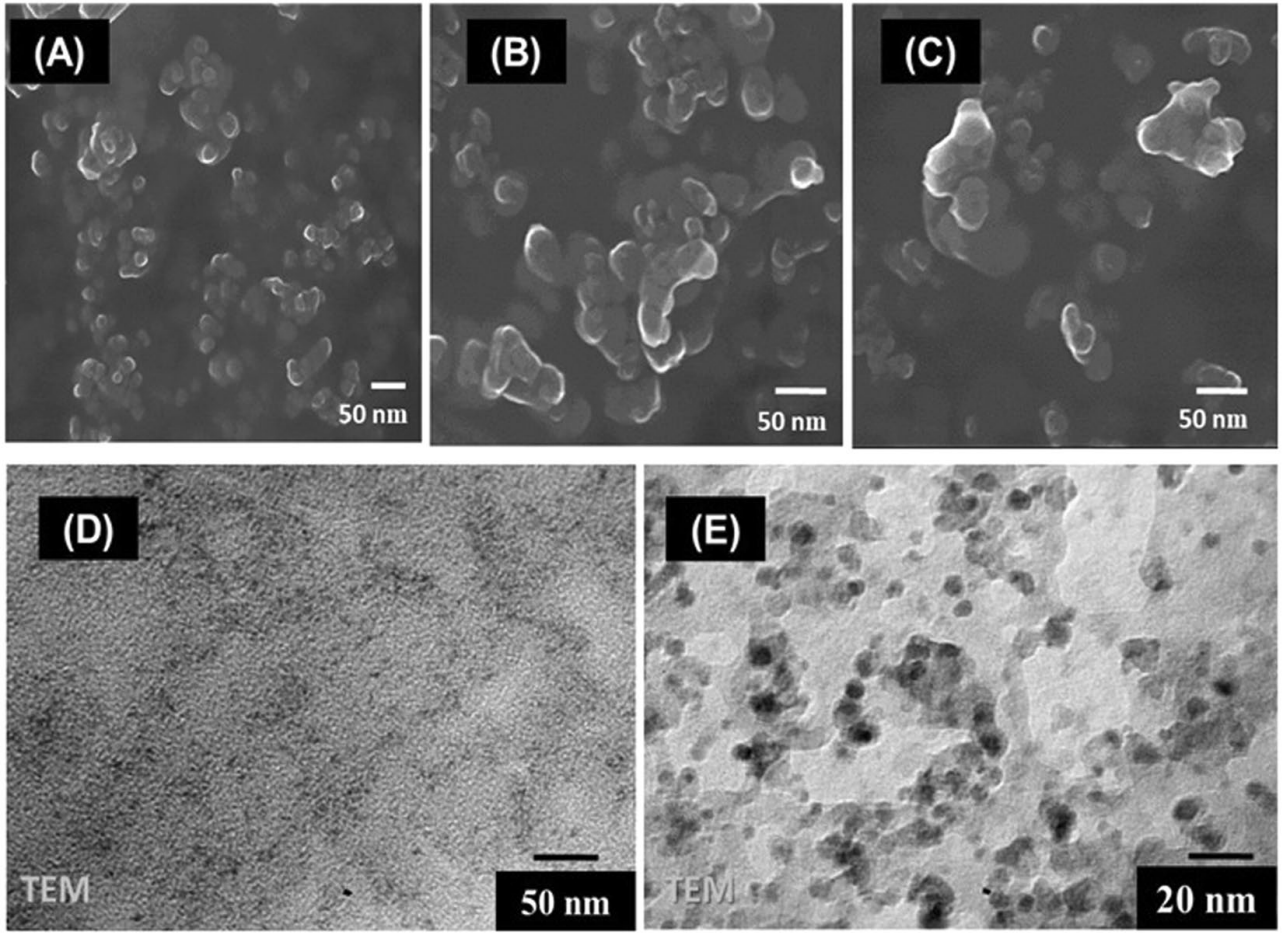
SEM images (top) of CdTe-MPA QDs suspended in ultra-pure water, (**A**) Raw QDs, (**B**) NIP-QDs and (**C**) MIP-QDs, and TEM images of raw CdTe-MPA QDs (**D**) and conjugated-QDs (**E**) in ultra-pure water.

In terms of size, TEM images revealed that raw QDs were homogeneously dispersed in aqueous solution, but tended to form aggregates (Fig. 3D–E). In DLS assays, the raw QDs had an average size of 4.1 nm in diameter (Figure S1). The Zeta potential revealed a homogeneous distribution of raw QDs in solution, with a mean value of −32.6 mV (Figure S2). This result is in agreement with the negative charge of raw QDs in PBS 10 mM, resulting from the carboxylic acid groups at their surface (pka 4.34, at 20 °C). SEM images were also collected but evidenced the formation of aggregates with an average size of 25 nm in diameter, which was inconsistent with the DLS and TEM data. This observation was probably linked to the sample preparation.

#### pH dependence

Concerning stability, several authors have addressed the importance of using a mercaptocarboxylic acid (MPA) in the synthesis of cadmium telluride QDs^48,49^. Of course, the use of a carboxylic acid as capping material implicated a pH dependent behavior. Accordingly, Yang and co-workers reported pH-dependent photoluminescence of QDs, being the fluorescent intensity of CdTe-MPA QDs optimal between pH (6–9)^50,51^. Herein, and considering that the main target of this work implicated the analysis of samples under near physiological conditions, all studies were conducted in PBS 10 mM (pH 7.4).

#### FTIR data

A typical FTIR spectrum of raw QDs is shown in Figure S3. Carboxylic groups are present in the form of carboxylate anions, given the basic pH conditions of the synthesis and the buffer used to conduct the studies (PBS 10 mM). These are signaled several times in the spectra of the raw QDs. The symmetric stretching of COO- are located at 1404 cm^−1^; and the peaks of asymmetric stretching at 1561 cm^−1^, as reported in^52,53^. The presence of OH stretching is also evidenced by the broad peak at ~3400 cm^−1^.

### Preliminary studies

Preliminary studies were conducted to evaluate the effect of Myo standards on a solution of raw CdTe-MPA QDs, suspended in PBS 10 mM. These results showed that the fluorescence intensity of raw QDs was gradually quenched with increasing concentrations of Myo standards. Myo alone was able to adsorb into the surface of the CdTe-MPA QDs, from a concentration of ~2 × 10^−6^ mol/L. The overall emission intensity of the CdTe-MPA QDs decreased by ~75% when a concentration of ~4 × 10^−4^ mol/L was present. This effect was however assigned to unspecific interactions with Myo, a multi-charged species with an overall negative charge under physiological pH. This effect was expected to be reduced by conjugating the QDs with a polymeric layer on the outer surface. Thus, MIP and NIP materials would also serve as a protecting shell to avoid unspecific binding from other sample components.

In terms of polymerization, several ratios of monomer/cross-linker were tested first against different concentrations of Myo, to identify the suitability of this approach. The (2:1) molar ratio (AAM: MBA) gave the best result, using 5.5 × 10^−5^ M (0.93 mg/ml) of Myo as the imprinting concentration (Table S1). The UV-Vis spectra of the raw and the conjugated-QDs in PBS 10 mM are shown in Figure S4. The peak at 415 nm demonstrates the existence of Myo in the supernatants of MIP-QDs after polymerization. To a certain extent, the disappearance of this peak from the MIP-QDs is followed to confirm the protein removal during the washing steps.

#### Size and shape

Compared to raw QDs, TEM images revealed a higher degree of aggregation after the polymerization (Fig. 3D,E). This may be explained by the extension of the polymerization reaction, which may contribute to the formation of an amalgam of QD material and polymer. It may be also explained by surface interactions established between the polymeric layer on the QDs. This aggregation was also observed by SEM analysis (Fig. 3A–C). In this, the aggregates revealed a more spherical shape, reaching ~50 nm in diameter.

#### FTIR

The polymerization process implicated the disappearance of the absorption bands of the OH group at ~3400 cm^−1^, coming from the MPA (Figure S3). These peaks were softened by the polymeric matrix around the QDs, or else moved to 3700 cm^−1^, typically related to unbounded (free) –OH. This later condition could occur due to the protective effect of the polymeric shell. In addition, the C=O band at ~1700 cm^−1^ in conjugated-NIPs and at 1660 cm^−1^ in conjugated-MIPs, can be attributed to the amide bonds of the polyacrylamide polymer.

### Surface versus Bulk Imprinting

The imprinting on given surface may be established by means of different approaches, being surface and bulk imprinting the most common. In surface imprinting, the target molecule is attached to the surface and the polymer is grown after around the target. In bulk imprinting, the target molecule is mixed with monomers and the polymerization takes place within this mixture. Both were tested herein, because each approach has their own advantages and disadvantages.

**Figure 4:**
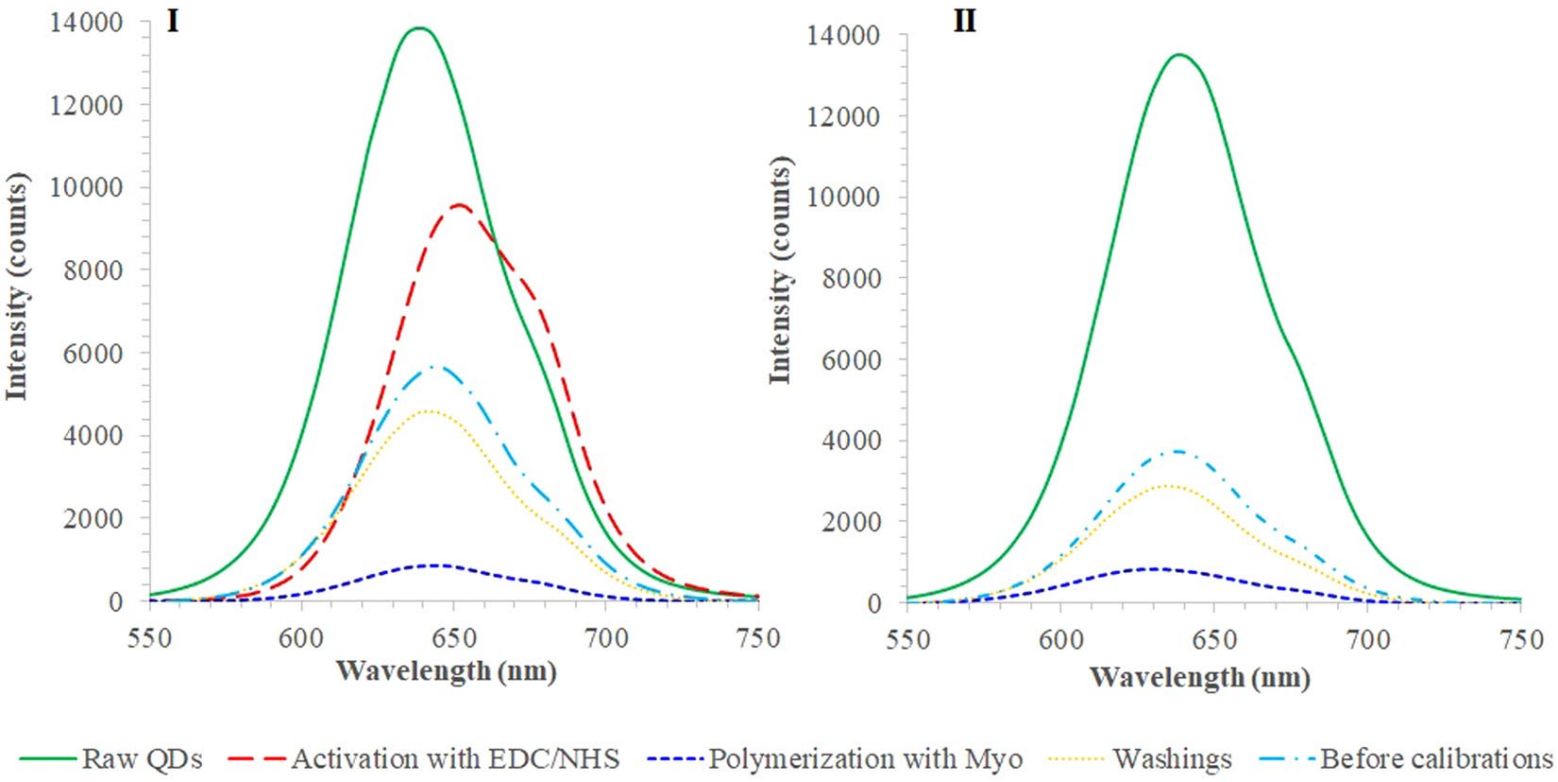
Fluorescence emission of MIP-QDs during assembly. (**I**) Surface imprinting, (**II**) bulk imprinting (PBS 10 mM).

Figure 4 shows the emission spectra of MIP-QDs prepared by surface imprinting (Fig. 4-I) and bulk imprinting (Fig. 4-II). In these, the different stages of the chemical modification of the QDs are followed in terms of fluorescence intensity. The first stage of QDs modification was made only on the surface imprinting approach, and consisted in the activation of the carboxylic groups. This was necessary to ensure that the polymer would be covalently bond to the QDs. The result of this modification was a fluorescence intensity decrease of about 30%. The subsequent stage was the imprinting, in which the polymer was formed in the presence of the protein. The fluorescence intensity was significantly blocked at this point, shifting almost to zero. The subsequent stage was the protein removal, made by several washing procedures, which recovered the fluorescence intensity. This recovery was much more significant in the surface imprinting (Fig. 4-I), meaning that part of the QDs surface was exposed again after Myo removal. The QDs were after stabilized in buffer before proceeding with the calibration.

### Effect of incubation time

The rebinding time (the time given for Myo to incubate with the QDs) is a critical step in MIP, and therefore it was evaluated. It was assessed for MIP-QDs prepared by surface imprinting, incubated in 1.5 nM Myo, and along 300 minutes. The results obtained are shown in Figure S5. In general, the MIP-QDs displayed a higher adsorption rate in the first 30 min, as the fluorescence signal decreased quickly before that (fluorescence intensity changed from ~11500 at time zero to ~7200 after 30 minutes incubation). In this time frame, the protein molecules could easily reach the imprinted cavities within the polymeric matrix, as these were mostly vacant. With the increasing time of Myo in solution, saturation of the surface imprinted cavities was expected to occur, followed by thermodynamic equilibrium and consequent stability of the fluorescent signal.

The corresponding control materials (NIP) were also tested in parallel. Testing these materials allowed identifying the dimension of the rebinding of Myo to the imprinted sites (only present in the MIP materials), compared to the rebinding of Myo to non-specific positions (only present in the NIP materials). As may be seen in Figure S5-B, the fluorescence changed more slowly and less intensely, never reaching an equilibrium pattern. This behavior confirmed the existence of non-specific interactions between Myo and the polymeric material, but these interactions were negligible in the first periods of incubation (fluorescence intensity changed from ~5700 to 4800 after 30 minutes) when compared to the MIP behavior.

Overall, the signal decayed in the first 30 minutes 37% and 16% in the MIP and NIP materials, respectively. The time that seems most suitable for incubation was 30 min. Still, as in point-of-care quick responses are often necessary, the system was also typified for a 5-minutes incubation period.

### Rebinding features

**Figure 5:**
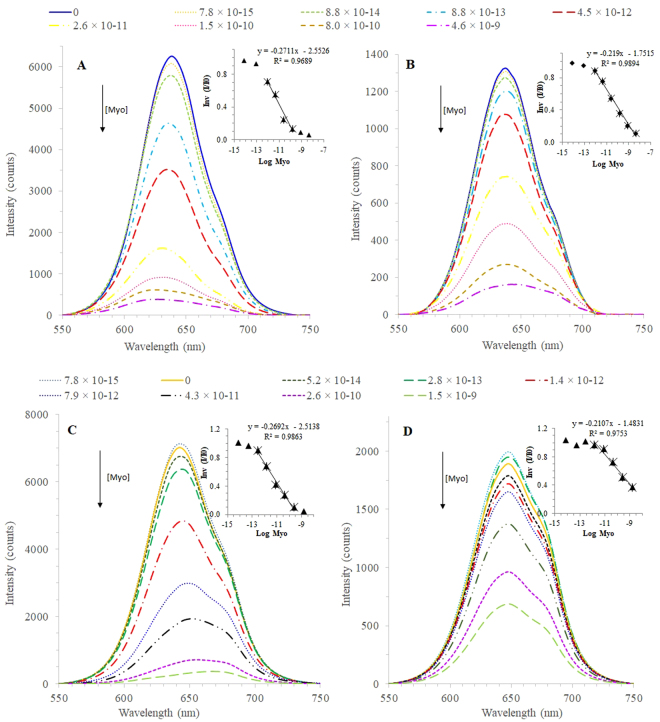
Fluorescence emission spectra of (**A**) MIP-QDs and (**B**) NIP-QDs prepared by bulk imprinting, and (**C**) MIP-QDs and (**D**) NIP-QDs prepared by surface imprinting, upon incubation of Myo standard solutions (PBS 10 mM; 5 minutes incubation). Inset: The correspondent Stern-Volmer plots.

**Figure 6:**
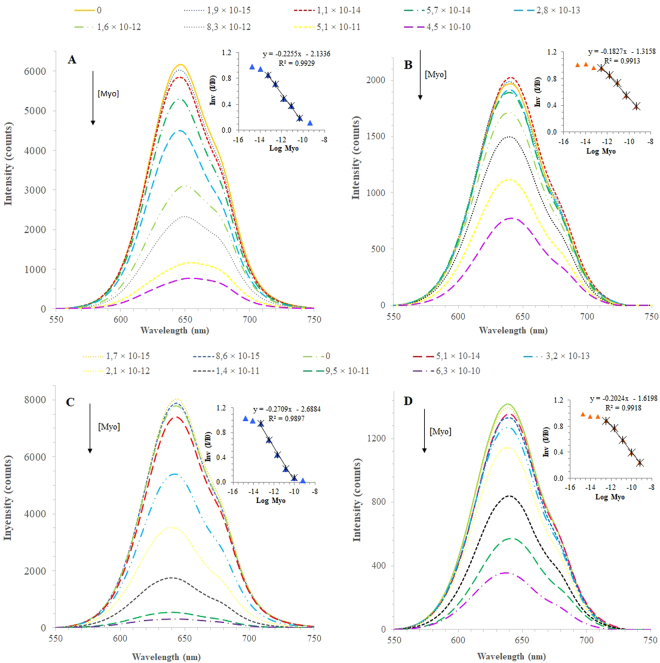
Fluorescence emission spectra of (**A**) MIP-QDs and (**B**) NIP-QDs prepared by bulk imprinting, and (**C**) MIP-QDs and (**D**) NIP-QDs prepared by surface imprinting upon incubation of Myo standard solutions (PBS 10 mM; 30 minutes incubation). Inset: The correspondent Stern-Volmer plots.

The rebinding features of the conjugated-QDs (MIP- and NIP-based) were evaluated in the presence of Myo standard solutions of increasing concentrations. The conjugated QDs used for this purpose were obtained by bulk and surface imprinting, and incubated in Myo for 5 or 30 minutes. For control purposes, the response of raw CdTe-MPA QDs incubated for 5 or 30 minutes was also assessed. The emission spectra obtained were plotted (Figs 5, 6 and S6) and the resulting analytical data was listed in Table S3.

The huge differences observed in this study were between MIP and NIP materials (as highlighted in Figure S7). Regardless the imprinting approach or the period of incubation, it was clear that the NIP-based QDs displayed a much lower fluorescence intensity values and their rate of decay upon Myo incubation was also slower than the corresponding MIP materials. This response confirmed that the dominant factor of the QDs fluorescence quenching was the rebinding of Myo to the imprinted sites generated by molecular imprinting. Although Stern-Volmer plots have in general higher slopes for the MIPs, these plots do not evidence clearly this huge difference in terms of sensitivity (Figs 5 and 6). Stern-Volmer plots handle relative data compared to the blank signal of the buffer, and disregard the huge difference of the absolute fluorescence intensity between the materials.

In general, MIP-QDs prepared by surface imprinting (Figs 5C and 6C) displayed better performance than the QDs prepared by bulk imprinting (Figs 5A and 6A). In general, the surface approach widened the linear range of response, by increasing the upper limit of linear range (ULLR) and/or decreasing the lower limit of linear range (LLLR). This was only valid for NIP materials and more significant for 30 minutes incubation (Table S3). The LOD of surface imprinted materials was also lower that the corresponding bulk materials, evaluated under the same conditions.

Regarding the time of incubation, the 30 minutes (Fig. 6) contributed mostly to decrease the concentration range of linear response, by which the LLLR was ~1-decade concentration lower. This was however valid for both MIP-QDs and NIP-QDs materials. This result was consistent with the previous study, described in section 3.4. Moreover, the response of the raw QDs was negligible within the concentration of interest, in which the MIP-QDs were able to promote a linear response.

Finally, the stability of the signal of the QD probes was evaluated throughout the time, upon resuspension of the obtained pellets in fresh PBS, 10 mM. This procedure was repeated for 5 days. The results obtained are shown in Figure S8. In general, the fluorescence intensity of the MIP- and NIP-based QDs did not change significantly within 5 days, thereby ensuring the reliability of the conjugated-QDs to be used as sensors for the calculation of myoglobin concentration in solution.

Overall, the best conditions were obtained for MIP-QDs, prepared by surface imprinting and incubated in each Myo concentration along 30 minutes. The corresponding LOD was 7.6 × 10^−15^ mol/L, and the concentration range of linear response was 5.06 × 10^−14^–9.50 × 10^−11^ mol/L. This material also displayed the highest IF factor, equal to 1.34 (Table S3), and excellent stability.

### Selectivity

Instead of testing the effect of individual species upon the modified QDs, the MIP materials were tested directly in the presence of synthetic human serum samples, as these hold an equal composition to real samples. First, the effect of the serum alone was tested by incubating the MIP-QDs in serum aliquots with different degrees of dilution. The more diluted the serum, the lowest the effect upon fluorescence. A dilution of 1000 times serum in PBS 10 mM, without further treatments, incubated for 30 minutes, rendered no obvious impact on the fluorescence signal (Figure S9).

The linear response of MIP-QDs (Figure S9A) started with lower concentrations of Myo standards and for a wider concentration range [(5.6 × 10^−14^–2.1 × 10^−10^) mol/L; (8.6 × 10^−13^–3.2 × 10^−9^) g/ml] comparatively to NIP-QDs [(2.9 × 10^−13^–1.1 × 10^−9^) mol/L; 4.5 × 10^−12^–1.7 × 10^−8^ g/ml] (Figure S9B). The calibration curves indicated that MIP-QDs remained more sensitive to Myo (IF = 1.05) in serum, with an LOD of 8.91 × 10^−15^ g/ml. The linear range of MIP-QDs was below the interval of concentrations of Myo associated to myocardial infarction [(9.52 × 10^−9^–4.71 × 10^−8^) g/ml; (5.6 × 10^−10^–2.77 × 10^−9^) M].

### Application to serum sample analysis

To test the possible use of the MIP-QDs in the analysis of real samples, 1000 ×  diluted synthetic human serum was spiked with a Myo concentration of 2.28 × 10^−12^ g/ml and then incubated for 30 minutes. The fluorescence readings were taken for both conjugated-QDs and the experimental errors were calculated from the calibration curve. The concentration determined of Myo in MIP-QDs was 1.83 × 10^−12^ g/ml (±19.9%). Taking in consideration that the synthetic serum used in this study is 1000-fold diluted, the concentrations of Myo obtained from the concentrated synthetic human serum was 1.83(±0.2) × 10^−9^ g/ml, which nears the cut-off levels of Myo in serum. Attending to the previous results, the MIP-QDs herein synthesized can be applied to determine concentrations of Myo in human serum, below the cut-off values reported for Myocardial Infarction occurrence (9.52 × 10^−9^–4.71 × 10^−8^) g/ml. Two more samples were analyzed similarly, considering two additional concentrations levels, of higher level and lying within the linear concentration range of the MIP-QDs. The overall values obtained have been listed in Table S4, also confirming the accuracy of the method.

## Conclusions

The conjugation of plastic antibodies and quantum dots was successfully established herein for Myo determination. The use of surface imprinting and longer incubation times seems to favor the analytical performance of the final material.

In addition, the imprinting of Myo on the QDs enabled the detection of Myo in lower concentration levels than by using the QDs alone. It is important to highlight that the interaction between Myo and unmodified QDs is non-specific, meaning that any other protein could lead to a similar response. Such response is probably due to ionic interactions established between the negative carboxylate functions and the positively charged amino acids located in the outer surface of the Myo 3D-structure. imprinting. A straight comparison to the NIP material also allowed to confirm that the MIP response was being dominated by rebinding to imprinted positions.

Overall, the conjugated-QDs used herein displayed attractive features, such as good dispersibility, high binding capacity and selectivity for Myo in complex serum matrices, when compared with the non-molecular imprinted counterparts. In addition, their long-term stability in aqueous solutions was also an advantage with rapid detection result, an important parameter to consider in the context of myocardial infarctions and point-of-care needs.

## Methods

### Materials

Solutions were prepared with ultrapure water (specific conductivity < 0.1 μS cm^−1^) and all chemicals were of analytical grade quality. Tellurium powder (200 mesh, 99.8%), sodium borohydride (NaBH_4_, 99%), cadmium chloride hemi-pentahydrate (CdCl_2._ 2.5H_2_O, 99%), acrylamide (AAM), *N*, *N*′- methylenebis(acrylamide) (MBA) and Myo were purchased from Sigma; 3-mercaptopropionic acid (MPA, 99%) and absolute ethanol (99.5%) were obtained from Fluka and Panreac respectively. *N*-Hydroxysuccinimide (NHS), 2-aminoethylmethacrylate hydrochloride (AEMH.HCl) and *N*-ethyl-*N*′-(3-dimethylaminopropyl) carbodiimide hydrochloride (EDC) were purchased from Merck, proteinase-K from AMRESCO and phosphate buffered saline (PBS) from Aldrich. Ammonium persulfate (APS) was acquired from Analar Normapur, tetramethylethylenediamine (TEMED) from TCI and human serum HN from PZ CORMAY S.A., Poland.

### Apparatus and analysis

Fluorescence measurements were performed on a Lumina Fluorescence Spectrometer (Thermo Scientific) equipped with a 150 W continuous wave xenon-arc lamp, as light source. Fluorescence emission spectra were acquired at 460 nm excitation wavelength, in the interval [550–750] nm, at a scanning rate of 600 nm/min. Both excitation and emission slit widths were adjusted to 20 nm, and the photomultiplier tube (PMT) voltage to 300 mV. Ultraviolet-visible (UV-vis) spectra were recorded on an Evolution 220 UV-visible spectrophotometer (Thermo Scientific). Fourier Transform Infrared (FTIR) spectra were collected using a Nicolet 6700 spectrometer coupled to an Attenuated Total Reflectance (ATR) sampling accessory of germanium contact crystal with a resolution of 32 cm^−1^ and a spectral range of 4000–700 cm^−1^. The morphological features and size distribution of all QD nanostructures were assessed via transmission electron microscopy (TEM), using bright-field and dark-field imaging, in a Hitachi H-9000-NA, operated at an accelerating voltage of 200 kV. For microscopy analysis, the powder samples were dispersed in deionized water, and applied dropwise onto a holey carbon layer, supported on a 300 Mesh copper grid. The copper grids used on the TEM observation were placed in a metallic support using a carbon adhesive tape. The surface morphology of all QD nanostructures were investigated using a field emission gun scanning electron microscope (FEG-SEM; JEOL-JSM7001F) operated at an accelerating voltage of 15 KV. Dynamic Light Scattering (DLS) and Zeta Potential analysis were performed on a Partica SZ100 series Nanoparticle Analyzer (HORIBA).

### Synthesis of sodium hydrogen telluride (NaHTe) precursor

The preparation of the precursor followed the methods commonly used in these assays but with some modifications. Briefly, 180 mg of sodium borohydride was transferred to a three-neck flask with 38.1 mg of tellurium powder and 5 ml of ultrapure water (previously saturated with N_2_). The flask was immediately immersed in an 80 °C water bath with continuous stirring and the reaction was allowed to proceed until consumption of the black powder and appearance of a purple solution of NaHTe. This solution was readily used in the following step.

### Synthesis of the capped cadmium telluride quantum dots (CdTe-MPA QDs)

Aqueous soluble QDs were prepared according to Zhou *et al*. with little modifications^54,55^. Briefly, CdCl_2_ (4.0 × 10^−3^ mol) was mixed with 3-mercaptopropionic acid (MPA) (6.8 × 10^−3^ mol) and 100 ml of ultrapure water in a three-necked flask. The pH was adjusted to 11.5 with 1 M sodium hydroxide added dropwise and the solution was further degassed with N_2_ for 30 minutes. The NaHTe aqueous solution was transferred into this flask at once and the obtained solution was refluxed. The molar ratio (Cd^2+^:Te^2-^:MPA) was optimized to (1:0.1:1.7). Different sized QDs were obtained by prolonging the refluxing time. The as prepared solutions were precipitated in absolute ethanol and the precipitates further separated by centrifugation at 4000 rpm, for 10 min, at room temperature. Finally, the powders were vacuum dried at room temperature, for several days. The resultant QDs were ground and stored in a desiccator protected from light and hereinafter referred to as raw QDs.

The optical properties of the raw QDs were controlled by UV/visible spectrophotometry assays. For this purpose, the diameter and extinction coefficient were determined by the equations,

1 $${\rm{D}}=(9.8127\times {10}^{-7}){\lambda }^{3}-(1.7147\times {10}^{-3}){\lambda }^{2}+(1.0064)\lambda -194.84$$

2 $${\rm{\varepsilon }}=3450\,{\rm{\Delta }}E{({\rm{D}})}^{2.4}$$

where *D* (nm) is the diameter of the nanoparticles, λ (nm) is the wavelength where the first excitonic absorption energy occurs, ΔE is the transition energy corresponding to that absorption (eV units) and $\varepsilon \,({\rm{L}}\times {{\rm{mol}}}^{-1}\times {{\rm{cm}}}^{-1})$ is the molar extinction coefficient of quantum dots, given by the Lambert-Beer’s Law^56^.

### Preparation of the conjugated-QDs (MIP-QDs)

Two strategies were followed to prepare the MIP- and NIP-QDs; i) surface imprinting (Fig. 1A) and bulk imprinting (Fig. 1B). Several combinations were tested during assembly of the MIP- and NIP-QDs (Table S1). For the surface imprinting strategy, the carboxylic groups at the surface of QDs were first modified by the active ester method, through reaction of 5.36 × 10^−6^ M of raw QDs solution in PBS 10 mM (pH 7.4) with freshly prepared EDC (1.59 × 10^−6^ M) and NHS (7.15 × 10^−6^ M) solutions, during 30 min (r.t.). The resultant solution was centrifuged and the supernatant discarded. Further, Myo solution (5.50 × 10^−5^ M) in PBS was incubated for 1 h (r.t.) and later centrifuged at 14680 rpm for 10 minutes. Pellets were re-dispersed in PBS 10 mM again. After, 2-aminoethyl methacrylate hydrochloride (AEMH.HCl) (1.51 × 10^−5^M) was added with 20 minutes stirring (r.t.) and the supernatants discarded again. Next, acrylamide (AAM) (5.30 × 10^−4^ M), *N*,*N*′-methylenebis(acrylamide) (MBA) (2.81 × 10^−4^ M), tetramethylethylenediamine (TEMED) (6.68 × 10^−6^ M) and ammonium persulfate (APS) (8.76 × 10^−6^ M) were added to the mixture and the solution degassed with N_2_ for 10 minutes. The polymerization reaction was continued for 1 h (r.t.). Both MIP- and NIP-QDs were centrifuged several times and washed with ethanol until no protein was detected in the supernatants of MIP-QDs by UV-Vis spectroscopy. The synthesis of NIP-QDs followed the same strategy but without protein incubation. For simplicity, MIP- and NIP-QDs will hereafter be referred as conjugated-QDs.

For the bulk imprinting strategy (Fig. 1B), the same conditions were used except that the previous activation of the carboxylic groups with EDC/NHS on the surface of the QDs was not performed. Instead, the protein was mixed in bulk with QDs and all the reagents involved in the polymerization step. Synthesis of NIP-QDs followed the same strategy but without the step of Myo incubation.

### Fluorescence measurements

All measurements were made in a quartz cuvette with 1 ml PBS 10 mM, and micro-volumes of Myo standards were added to the reaction vessel. Fluorescence spectra were acquired after incubations of Myo standards at room temperature. The Stern-Volmer method (equation 3) and the calculation of imprinting factor (IF) were made to assess and compare the prepared materials (equation 4).

3 $${{\rm{F}}}_{0}/{\rm{F}}=1+{K}_{{\rm{S}}{\rm{V}}}[{\rm{Q}}]$$

4 $${\rm{IF}}=({K}_{\mathrm{SV},\mathrm{MIP}}/{K}_{\mathrm{SV},\mathrm{NIP}})$$

F and F_0_ are the fluorescence intensity of QDs in the presence and in the absence of protein, respectively; *K*_SV_ is the Stern -Volmer constant and [Q] is the quencher concentration in solution. The LOD was calculated as the concentration of Myo that quenched three times the standard deviation of the blank signal.

### Tests in spiked synthetic human serum

To evaluate the effect of other interfering proteins on conjugated-QDs, calibrations were made in a 1000-fold diluted synthetic human serum, in PBS 10 mM. In parallel, both conjugated-QDs were suspended separately in 1 ml of this serum and spiked with a specific concentration of Myo standard. Single fluorescence readings were performed to evaluate the selectivity of the conjugated-QDs (MIP- and NIP-QDs).

### Data availability

All data generated or analysed during this study are included in this published article (and its Supplementary Information files).

## Electronic supplementary material

Supplementary data
