# Supplementary material for: Plastic antibodies tailored on quantum dots for an optical detection of myoglobin down to the femtomolar range
Source: Sci Rep. 2018 Mar 21;8:4944. doi: 10.1038/s41598-018-23271-z (PMC5862838; doi:10.1038/s41598-018-23271-z)
Supplement: Supplementary file 1 — Supplementary data [file 41598_2018_23271_MOESM1_ESM.pdf]

Supplementary Material

to

Plastic antibodies tailored on quantum dots for an optical detection of myoglobin down to the femtomolar range

Ana Margarida Piloto[1)], David S. M. Ribeiro[2)], S. Sofia M. Rodrigues[2)], C. Santos[3,4)], João L. M. Santos[2)], M. Goreti F. Sales[1)*]

[1]BioMark /ISEP, School of Engineering of the Polytechnic Institute of Porto, Porto, Portugal

[2]LAQV/REQUIMTE, Faculty of Pharmacy of Porto University, Porto, Portugal.

[3]EST Setúbal, CDP2T, Instituto Politécnico de Setúbal, Setúbal, Portugal

[4]CQE, Instituto Superior Técnico, Universidade de Lisboa,Lisboa, Portugal

Table S1. Polymerization conditions used for the synthesis of the conjugated-QDs, after 1 h Myo imprinting by surface in PBS 10 mM.

Combination	AAM (M)	MBA (M)	Molar ratio (AAM:MBA)/ Myo	Myo (M)
1	7.42×10^{-4}	1.63×10^{-4}	(5:1)	7.03×10^{-5}<br>5.50×10^{-5}<br>1.21×10^{-5}<br>5.90×10^{-6}
2	5.30×10^{-4}	2.81×10^{-4}	(2:1)	7.03×10^{-5}<br>5.50×10^{-5}*<br>1.21×10^{-5}<br>5.90×10^{-6}
3	1.06×10^{-4}	9.73×10^{-5}	(1:1)	7.03×10^{-5}<br>5.50×10^{-5}<br>1.21×10^{-5}<br>5.90×10^{-6}

*best analytical performance for MIP-QDs.

Table S2. Physical parameters of raw CdTe-MPA QDs in ultra-pure water.

Particle size diameter *D* (nm)	Molar extinction coefficient, ε (L mol^{-1} cm^{-1})	Concentration (mol/L)	λ_{abs} (nm)	λ_{em} (nm)
2.20	57295	1.52×10^{-6}	495	544
2.48	74641	1.85×10^{-6}	505	559
2.90	104797	1.14×10^{-6}	525	595
3.66	160733	6.50×10^{-7}	600	658

The diameter, D, calculation was supported by the following reference:

Yu, W.W., Qu, L.H., Guo, W.Z., Peng, X.G., 2003. Experimental determination of the extinction coefficient of CdTe, CdSe, and CdS nanocrystals. Chem Mater 15(14), 2854-2860.

Table S3. Analytical parameters determined upon incubation of Myo standards in PBS 10mM.

**Analytical Feature**	**MIP-QDs**				**NIP-QDs**				**Raw QDs**	
Imprinting strategy	Bulk	Surface	Bulk	Surface	Bulk	Surface	Bulk	Surface	—	—
Incubation (min.)	5	5	30	30	5	5	30	30	5	30
LLLR (mol/L)	8.78×10^{-13}	2.77×10^{-13}	5.68×10^{-14}	5.06×10^{-14}	8.78×10^{-13}	1.40×10^{-12}	2.83×10^{-13}	3.24×10^{-13}	1.95×10^{-10}	2.42×10^{-11}
ULLR (mol/L)	1.52×10^{-10}	2.58×10^{-10}	5.10×10^{-11}	9.50×10^{-11}	4.57×10^{-9}	1.54×10^{-9}	4.53×10^{-10}	6.33×10^{-10}	4.47×10^{-8}	4.27×10^{-9}
LOD (mol/L)	9.19×10^{-14}	8.15×10^{-14}	1.22×10^{-14}	7.55×10^{-15}	2.58×10^{-13}	1.66×10^{-12}	2.07×10^{-13}	1.39×10^{-13}	3.76×10^{-11}	1.68×10^{-12}
Imprinting factor	1.24	1.28	1.23	1.34	—	—	—	—	—	—
Stern Volmer (K_{SV})	0.271	0.269	0.225	0.271	0.219	0.211	0.183	0.202	0.234	0.259

ULLR: Upper Limit of Linear Range; LLLR: Lower Limit of Linear Range; LOD: Limit of Detection.

Dynamic light scattering analysis

The measurements conditions for size are:

Dispersant: ultra-pure water

Measurement mode: nano Analysis Mode

Scan settings: Noise Cut Set

Run Duration: 240s

ND Filter: Auto

Detector position: Auto

Calculation: Standard Monodisperse

The DLS analysis resultant from low sampling time (320ns) with the size distribution computed in number base distribution is depicted in figure S1.

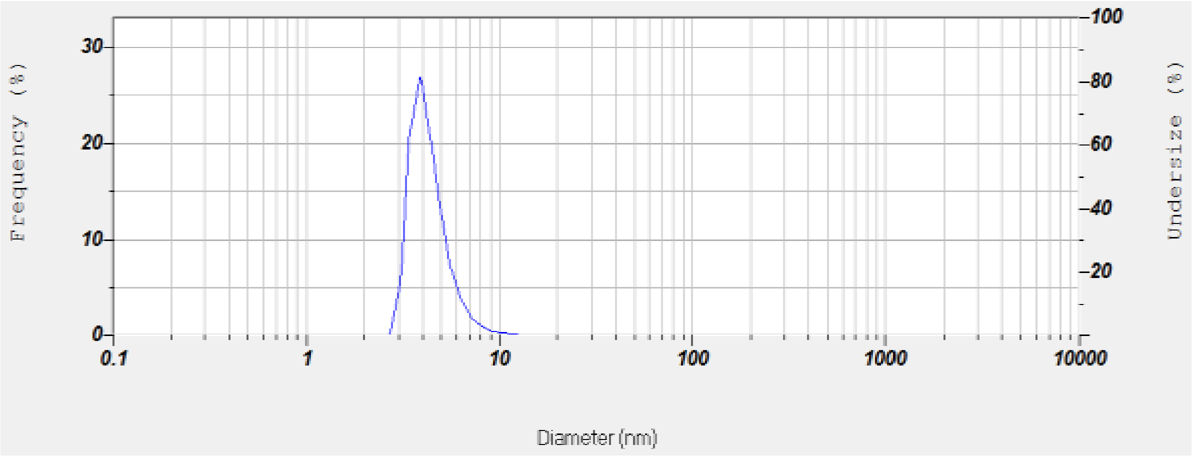

Figure S1. DLS spectra of red raw QDs in ultra-pure water. The average size of the particles is estimated in 4.1 nm.

Zeta potential (ζ) analysis

Diluted solutions of red raw QDs were used to determine the zeta potential. A homogeneous distribution of raw-QDs sizes was evidenced, according to the zeta potential distribution. The value of −32,6mV obtained confirmed the negative charged surface of the QDs.

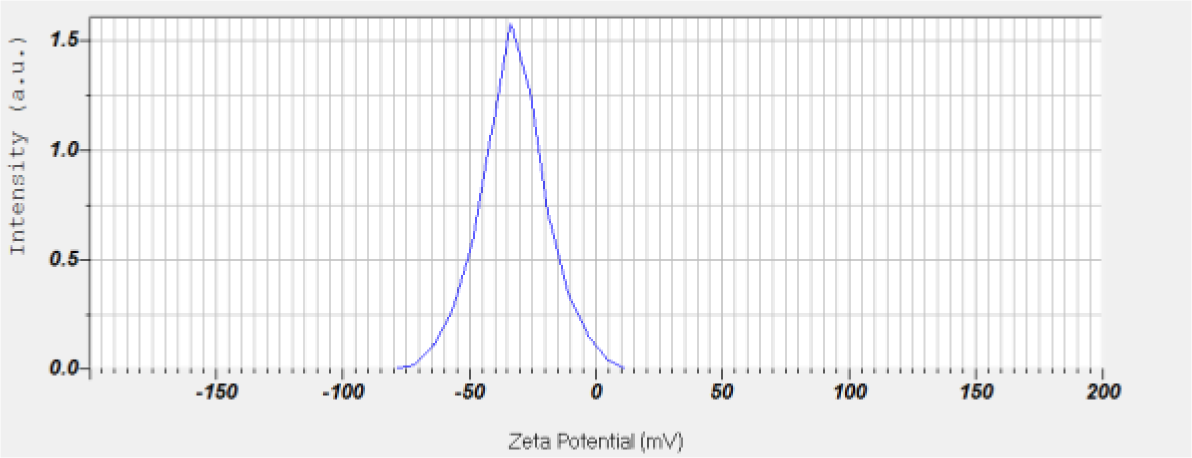

Figure S2. Zeta potential distribution of red CdTe-MPA QDs in ultra-pure water.

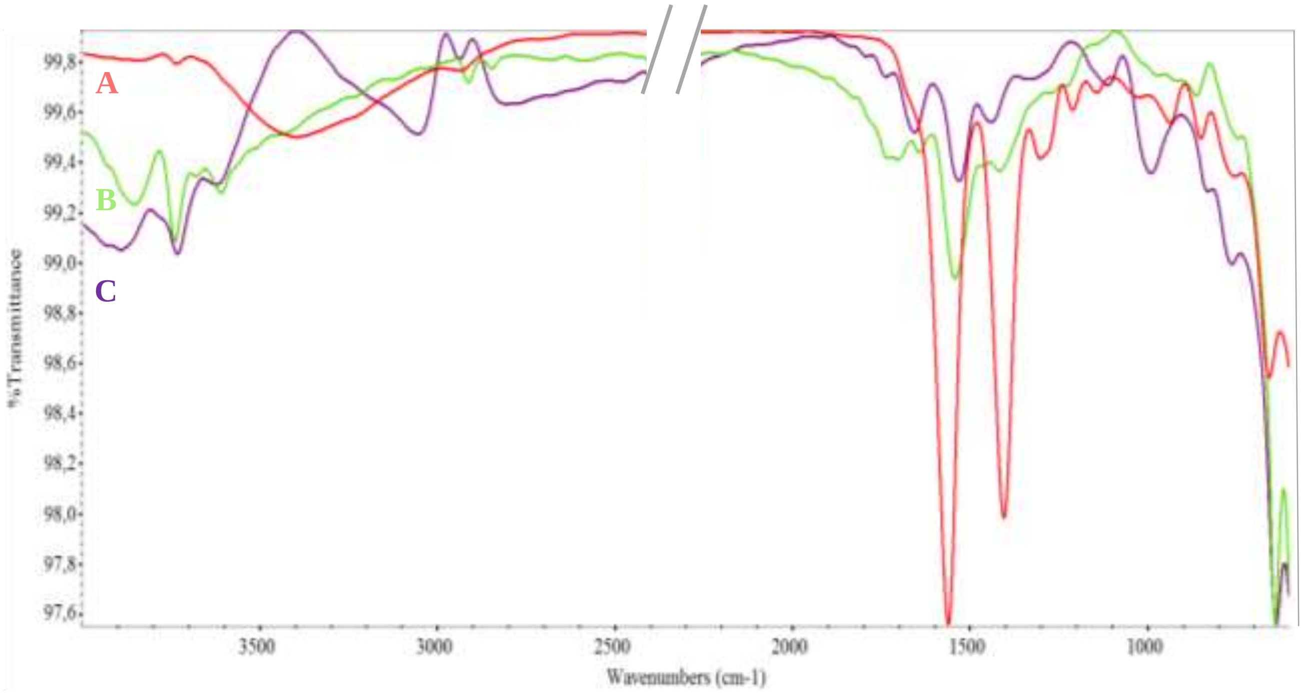

Figure S3. FTIR spectra of (**A**) raw CdTe-MPA QDs, (**B**) MIP-QDs and (**C**) NIP-QDs.

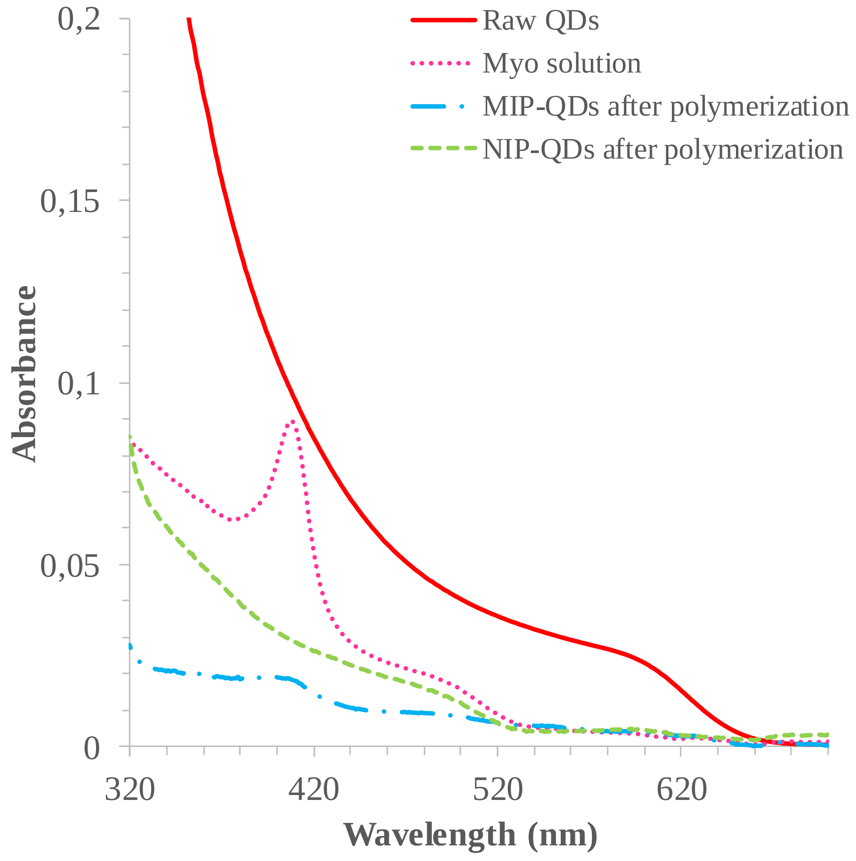

Figure S4. UV-Vis absorption spectra of the myoglobin imprinting solution and of the CdTe-MPA QDs suspended in PBS 10mM.

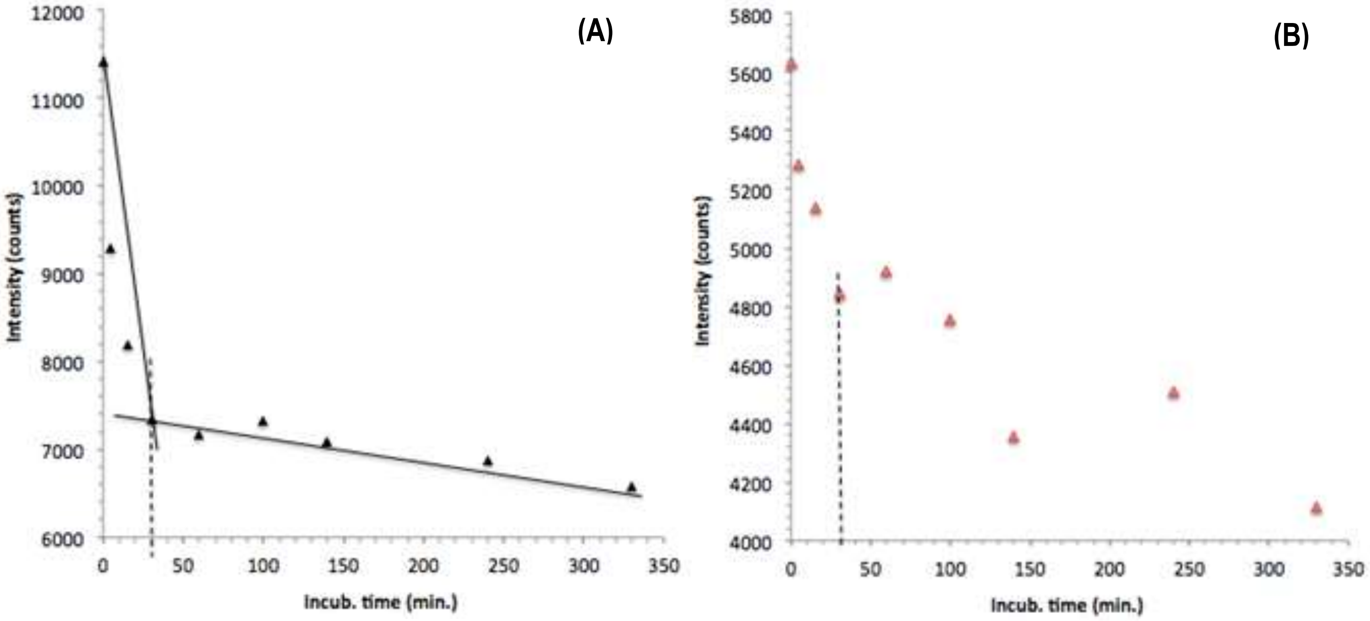

Figure S5. Adsorption kinetics of a Myo standard solution 1.5 nM, with increasing incubation times. (**A**) MIP-QDs and (**B**) NIP-QDs, prepared by surface imprinting strategy (PBS 10mM, r.t.).

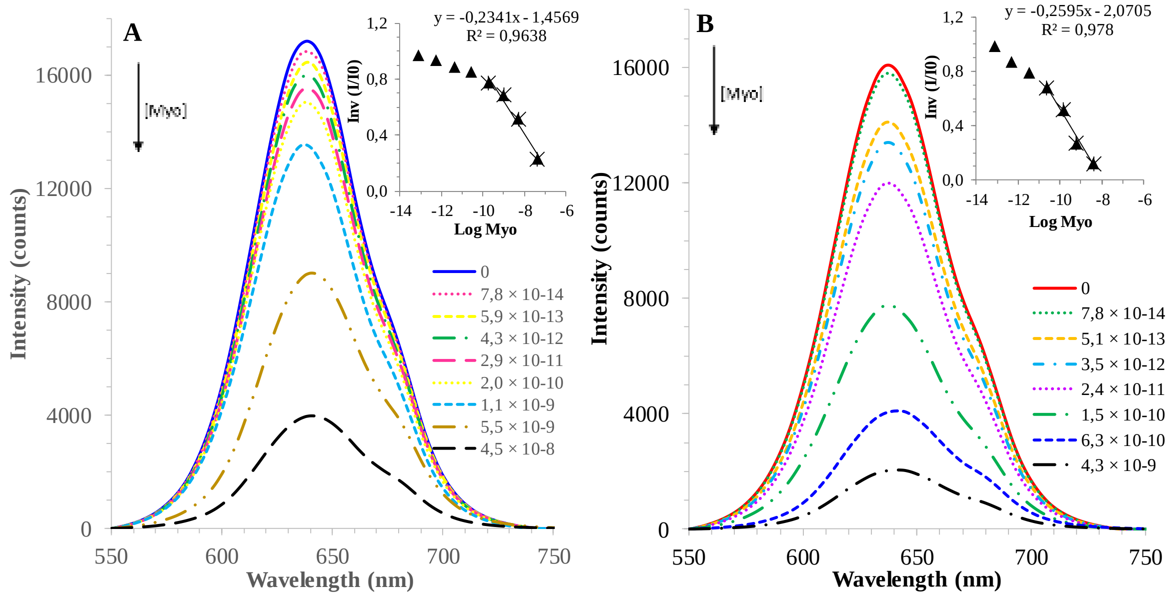

Figure S6. Fluorescence emission spectra of raw QDs, with addition of increasing concentrations of Myo standards. (**A**) after 5 min incubation; (**B**) after 30 min incubation, in PBS 10mM. Inset: The correspondent Stern-Volmer plot.

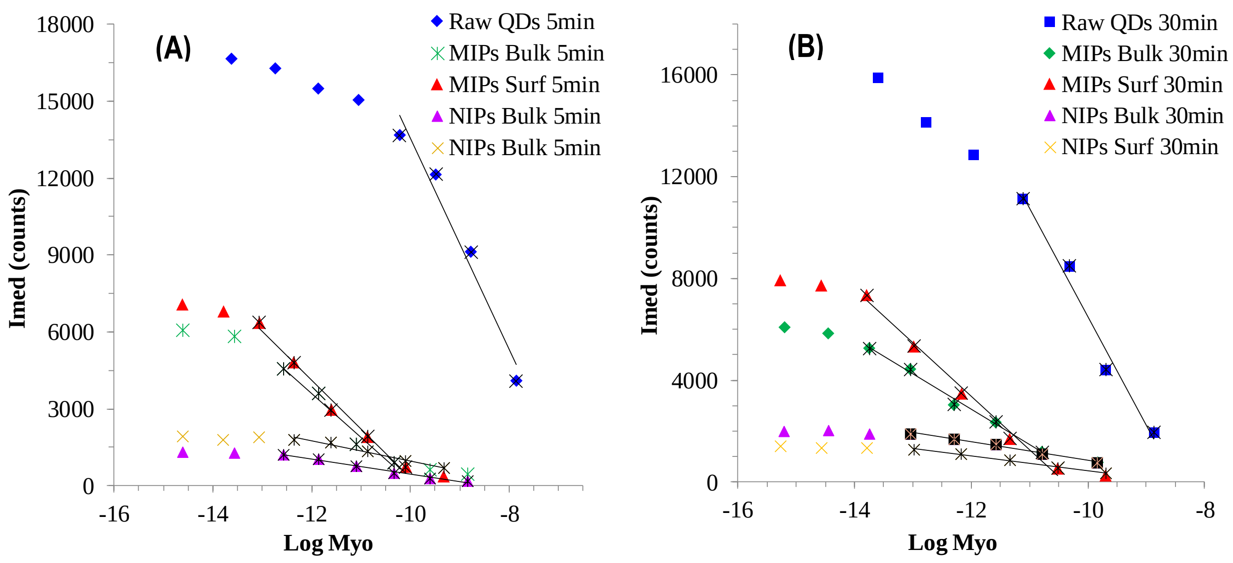

Figure S7. Comparative performances of raw QDs and conjugated-QDs using bulk and surface imprinting strategies, after incubation with Myo standards in PBS 10mM, for 5 min (left) and 30 minutes (right).

A

B

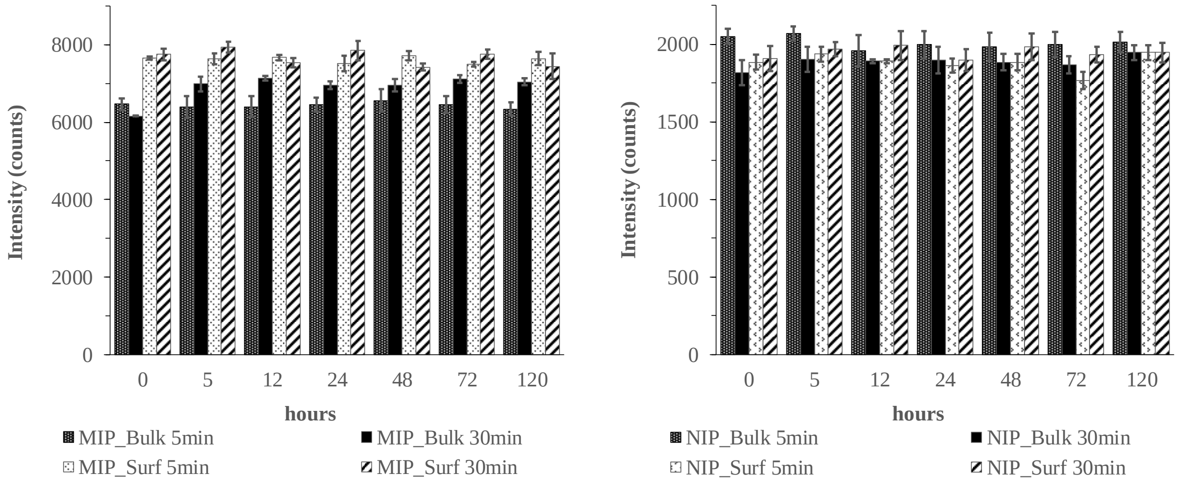

Figure S8. Fluorescence stability of the as-prepared MIP-based QDs (A) and NIP-based QDs (B), prepared by several imprinting strategies, prior to myoglobin standards calibrations, upon 5 days storage at 4ºC.

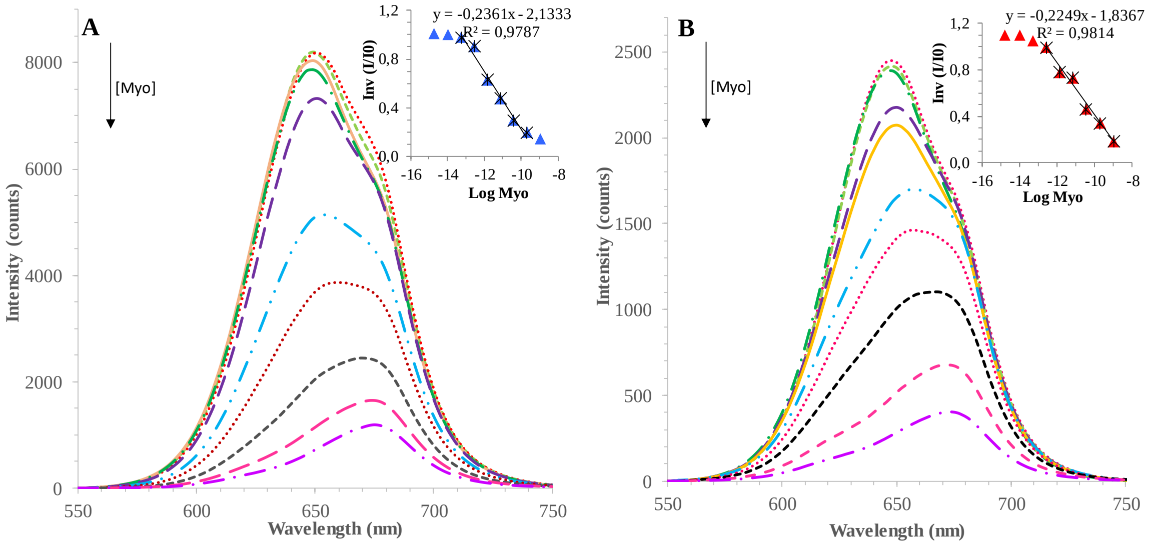

Figure S9. Fluorescence emission spectra from the selectivity test of MIP-QDs (A) and NIP-QDs (B), prepared by surface imprinting, with addition of increasing concentration of Myo standards (30 min incubation), in a 1000-fold diluted synthetic human serum sample. Inset: The correspondent Stern-Volmer plots.

Table S4. Spiked concentrations of Myo obatined with MIP- or NIP-based QDs, in a 1000× diluted serum (experimental errors indicated in %).

Spiked myoglobin concentration (g/ml)	Calculated myoglobin concentration (g/ml)	
	MIP	NIP
2.28×10^{-12}	1.83×10^{-12} ± 19.9%	4.66×10^{-13} ± 79.5%
1.60×10^{-10}	1.96×10^{-10} ± 22.6%	3.92×10^{-11} ± 75.4%
2.48×10^{-9}	2.77×10^{-9} ± 11.6%	5.47×10^{-10} ± 78.0%
